# Intraoperative management of pre‐operative flexion contractures using robotic‐assisted total knee arthroplasty

**DOI:** 10.1002/jeo2.70834

**Published:** 2026-07-16

**Authors:** Yugo Morita, Jonggu Shin, Mohammed El‐Hassan, Ittai Shichman, William J. Long, Peter K. Sculco

**Affiliations:** ^1^ Complex Joint Reconstruction Center, Adult Reconstruction and Joint Replacement Service, Hospital for Special Surgery New York New York USA

**Keywords:** distal femoral resection, flexion contracture, gap balancing, joint line restoration, robotic‐assisted total knee arthroplasty

## Abstract

**Purpose:**

To determine how the severity of preoperative flexion contracture influences intraoperative resection strategy, final balance, postoperative range of motion and Knee Injury and Osteoarthritis Outcome Score for Joint Replacement (KOOS‐JR) outcomes in robotic‐assisted total knee arthroplasty (RA‐TKA).

**Methods:**

This retrospective cohort study included primary imageless RA‐TKAs performed using the RObotic Surgical Assistant (ROSA) Knee System. Knees with preoperative flexion contracture were categorized as >0° to 5°, >5° to 10° and >10°. Preoperative standing full‐length radiographs were used to measure hip–knee–ankle angle. Intraoperative resection, gap and balance data were collected. Preoperative and 6‐week postoperative range‐of‐motion data were obtained from medical records, and KOOS‐JR scores were collected preoperatively and at 1 year.

**Results:**

In total, 263 knees with preoperative flexion contracture were included, with final intraoperative outcomes available for 227 knees. Distal medial femoral resection increased with contracture severity (9.0 ± 1.3, 9.3 ± 1.2 and 9.8 ± 0.8 mm in the >0°–5°, >5°–10° and >10° groups; *p* < 0.001), whereas tibial construct thickness remained similar (*p* = 0.908). Final gaps, gap imbalances and coronal laxity were comparable across groups. At 6 weeks, extension differed across groups (*p* = 0.030), whereas flexion did not (*p* = 0.138); residual flexion contracture >5° was observed in 2.5%, 0.0% and 10.3% of knees (*p* = 0.024). Among knees with paired 1‐year KOOS‐JR data (*n* = 137), preoperative, postoperative and change scores did not differ across groups (*p* = 0.336, *p* = 0.696 and *p* = 0.231). Each additional degree of flexion contracture was associated with greater distal medial femoral resection (*β *= 0.06 mm per degree, *p* = 0.009).

**Conclusions:**

Preoperative flexion contracture was associated with minor differences in intraoperative resection strategy, particularly distal medial femoral resection. Final intraoperative balance and early outcomes were similar across flexion contracture groups.

**Level of Evidence:**

Level III, retrospective cohort study.

**Abbreviations**
ANOVA: analysis of variance
CI: confidence interval
CR: cruciate‐retaining
HKA: hip–knee–ankle
KOOS‐JR: Knee Injury and Osteoarthritis Outcome Score for Joint Replacement
PCL: posterior cruciate ligament
PS: posterior‐stabilized
RA‐TKA: robotic‐assisted total knee arthroplasty
ROSA: RObotic Surgical Assistant
TKA: total knee arthroplasty

## INTRODUCTION

Preoperative flexion contracture is common in patients undergoing total knee arthroplasty (TKA) and ranges from mild limitation to severe fixed deformity [1, 26]. It is also clinically important, as it increases the risk of residual postoperative flexion contracture, which is associated with gait abnormalities, including reduced walking speed, increased energy expenditure, greater pain and inferior functional outcomes [18]. Persistent flexion contracture of ≥10° is linked to worse functional outcomes and lower patient satisfaction [20], while contractures exceeding 15° at 3 months postoperatively are likely to persist long‐term [17].

One strategy for improving extension is additional distal femoral resection, which increases the extension gap [16, 23]. However, increasing distal resection elevates the femoral joint line and may adversely affect mid‐flexion stability and coronal plane laxity [5, 7, 15]. Correction of flexion contracture during TKA is multifactorial and may involve osteophyte removal, posterior capsular release, selective soft‐tissue balancing, tibial resection and tibial slope adjustment, in addition to distal femoral resection [16, 23]. Robotic‐assisted TKA (RA‐TKA) provides a standardized workflow in which bone resections, alignment targets and intraoperative gap measurements are recorded objectively, offering a useful framework to evaluate how these technical adjustments relate to flexion contracture severity. In contrast, quantifying the relative contributions of femoral resection, tibial resection, tibial slope adjustment and insert thickness selection is challenging in conventional instrumented TKA [27].

Although prior studies have examined clinical outcomes of TKA in knees with flexion contracture, less is known about how contracture severity influences intraoperative resection strategy and final balance metrics within a robotic workflow. Therefore, this study aimed to determine how the severity of preoperative flexion contracture influences bone resection strategy and final intraoperative balance metrics in RA‐TKA. In addition, early Knee Injury and Osteoarthritis Outcome Score for Joint Replacement (KOOS‐JR) outcomes were evaluated, and the continuous association between flexion contracture severity and distal femoral resection, as well as clinical outcomes, was quantified. It was hypothesized that greater preoperative flexion contracture would be associated with modest differences in resection strategy, whereas final intraoperative balance would remain broadly similar across contracture groups.

## METHODS

### Study design and cohort

A retrospective cohort study was performed of consecutive primary robotic‐assisted total knee arthroplasties (RA‐TKAs) conducted by a fellowship‐trained surgeon at a high‐volume arthroplasty center between August 2022 and October 2024. Procedures using an imageless robotic system workflow were identified. Inclusion criteria were (1) primary RA‐TKA and (2) availability of preoperative standing full‐length lower‐extremity radiographs (either a low‐dose, biplanar, full‐body X‐ray imaging system [EOS] or a conventional long‐leg radiograph) permitting measurement of the mechanical hip–knee–ankle (HKA) angle. Exclusion criteria were (1) use of a constrained implant, including constrained condylar knee or rotating‐hinge constructs and (2) TKA performed for post‐traumatic deformity.

**Figure 1:**
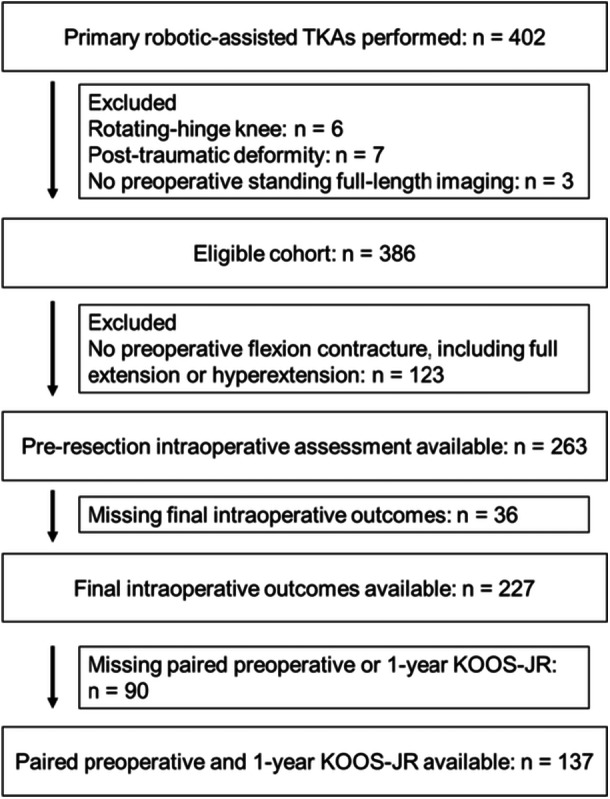
Flow diagram of cohort selection and data availability. Of 402 primary robotic‐assisted TKAs performed during the study period, 386 knees met the general eligibility criteria. After excluding knees without preoperative flexion contracture, 263 knees were included in the present analysis. Final intraoperative outcomes were available for 227 knees and paired preoperative and 1‐year postoperative KOOS‐JR scores were available for 137 knees. KOOS‐JR, Knee injury and Osteoarthritis Outcome Score for Joint Replacement; TKAs, total knee arthroplasties.

During the study period, 402 primary RA‐TKAs were performed. Of these, three lacked preoperative standing full‐length imaging, six involved rotating‐hinge implants and seven were performed for post‐traumatic deformity, leaving 386 eligible knees. For the present analysis, only knees with preoperative flexion contracture were included; knees with full extension or hyperextension were excluded. This yielded 263 knees with preoperative flexion contracture. Final intraoperative outcomes were available in 227 knees with complete measurements. Patient‐reported outcomes, consisting of paired preoperative and 1‐year postoperative KOOS‐JR data, were available for 137 knees (Figure 1).

### Surgical techniques

**Figure 2:**
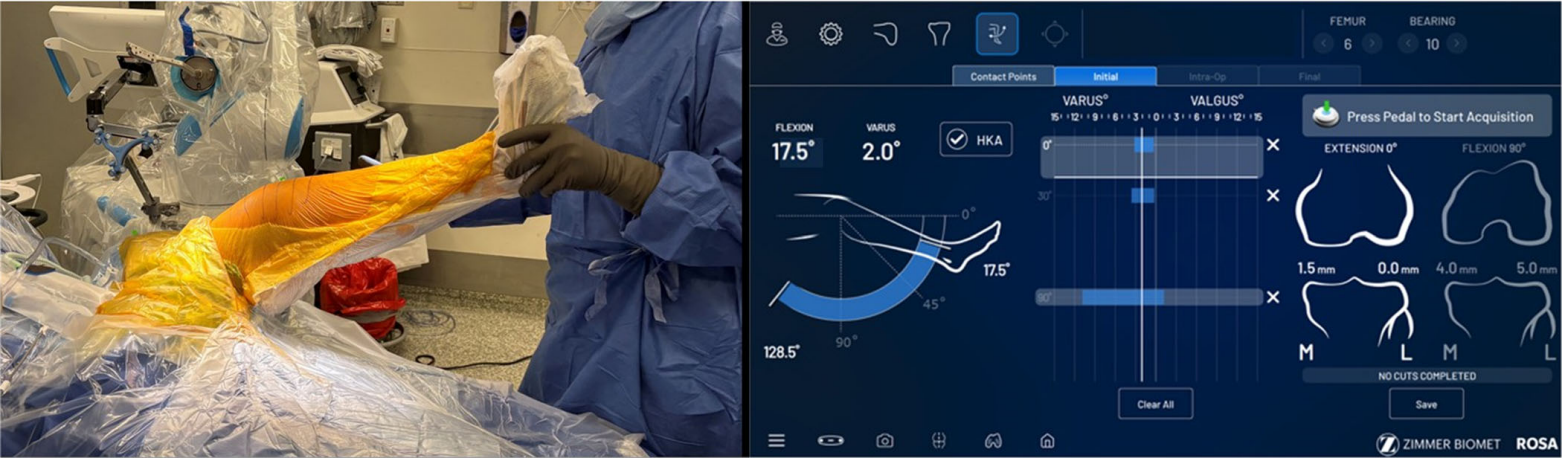
Representative intraoperative photograph and robotic system screen illustrating the assessment of flexion contracture under anaesthesia during imageless robotic‐assisted total knee arthroplasty. Passive knee range of motion was recorded after registration and before any bone resection. Flexion contracture was defined as the maximum extension angle measured during the initial robotic assessment under maximum manual correction.

All procedures were performed using an imageless Robotic Surgical Assistant (ROSA) Knee workflow (Zimmer Biomet) through a standardized medial parapatellar approach. Femoral and tibial trackers were secured with percutaneous pins, and anatomical landmarks were registered according to the manufacturer's protocol. Following registration, the passive range of motion under anaesthesia was recorded by the robotic system. Flexion contracture was defined as the maximum extension angle measured during the initial intraoperative assessment under maximum manual correction, prior to any bone resection (Figure 2). Medial and lateral compartment space (mm) were recorded in extension and at approximately 90° of flexion by the robotic platform. In addition, a standardized surgeon‐applied varus–valgus stress examination was performed in extension and at 90° of flexion, during which varus and valgus angular excursion (degrees) were recorded.

Alignment planning and balancing were performed within a standardized workflow. Varus knees were managed using a restricted kinematic alignment strategy adjusted according to measured laxity, whereas valgus knees were managed using an adjusted mechanical alignment strategy on the femoral side combined with a neutral tibial coronal resection. Tibial slope was targeted to the native slope, up to 8°. These parameters were incorporated into intraoperative planning of bone resections to achieve balanced extension and flexion gaps without excessive coronal laxity. Gap targets were not strictly symmetric. In varus knees, the lateral compartment was generally targeted to be slightly looser than the medial compartment in flexion, whereas in valgus knees, the medial side was typically maintained slightly tighter to avoid residual laxity.

Distal femoral and proximal tibial resections were performed under robotic guidance using a cutting guide, and coronal and sagittal resection parameters were recorded. Extension balance was then assessed, with selective soft‐tissue release performed as required. No formal medial collateral ligament (MCL) release or pie‐crusting was performed in varus knees; limited deep MCL release was performed only as part of the standard medial exposure. Once extension balance had been achieved, flexion balance was reassessed at 90° of flexion, and posterior femoral resections were completed. Following femoral preparation, posterior femoral osteophytes were removed, and limited posterior capsular release was performed when indicated. Trial components were inserted, and balance was reassessed in extension and at 90° of flexion before final implantation.

Implant design, either cruciate‐retaining (CR) or posterior‐stabilized (PS), was selected intraoperatively according to ligamentous stability and balancing requirements. In CR knees, the posterior cruciate ligament (PCL) was preserved and only partially released when excessive posterior femoral rollback or anterior lift‐off of the trial insert was observed in flexion. In PS knees, the PCL was resected. After trial implantation, stress examinations and gap measurements were repeated, and final gaps and coronal laxity metrics were recorded as the definitive intraoperative values [3].

Flexion contracture severity was categorized as >0°–5°, >5°–10° and >10° [6, 17, 23]. A compartment‐specific tibial‐side offset was calculated for the medial and lateral compartments as the combined tibial construct thickness (polyethylene insert plus tibial baseplate, mm) minus the corresponding validated proximal tibial resection depth (mm). Construct thickness reflected the total tibial‐side thickness (e.g., 10 mm) used intraoperatively and included both polyethylene and baseplate components [10, 22].

### Radiographic assessment

Preoperative coronal alignment was measured on standing full‐length radiographs (EOS or conventional long‐leg radiographs) as the mechanical HKA angle, with varus defined as positive and valgus as negative. For subgroup analyses, knees were categorized as varus (≥3°), neutral (−3° to 3°) or valgus (≤−3°) [25].

### Data analysis

Continuous variables are presented as mean ± standard deviation, and categorical variables as counts (percentages). Normality of continuous variables was assessed visually using histograms and *Q*–*Q* plots. Based on these distributions and the sample size, parametric methods were considered appropriate. The three flexion contracture groups (>0°–5°, >5°–10° and >10°) were compared using one‐way analysis of variance (ANOVA), with Welch's ANOVA applied when heterogeneity of variance was suspected. When post hoc pairwise comparisons were required, Tukey's honestly significant difference test or the Games–Howell test was applied as appropriate. Categorical variables were compared using the chi‐square test or Fisher's exact test when expected cell counts were small. Manipulation under anaesthesia within 1 year for stiffness and residual flexion contracture >5° at 6 weeks were also compared across groups. Statistical significance was set at a two‐sided *p* < 0.05, and no adjustment for multiple comparisons was performed. Missing data were not imputed. Analyses requiring final intraoperative resection, gap, coronal laxity or coronal resection‐angle measures were conducted in knees with complete final intraoperative measurements (*n* = 227), as these variables were unavailable in the exported robotic record for some cases. Preoperative and 6‐week postoperative extension and flexion angles were obtained from the medical records. KOOS‐JR scores were assessed preoperatively and at 1 year postoperatively (defined as 9–15 months according to registry conventions). Preoperative, 1‐year postoperative and change in KOOS‐JR scores (postoperative minus preoperative) were analysed in knees with paired KOOS‐JR data (*n* = 137). As additional analyses, multivariable linear regression models adjusted for age, sex, BMI, implant type and preoperative HKA angle were used to evaluate the association between flexion contracture group and each continuous intraoperative and clinical outcome. A separate linear regression model evaluated the continuous association between flexion contracture severity and distal medial femoral resection among knees with preoperative flexion contracture. Results from regression models are reported as regression coefficients with 95% confidence intervals (CIs). All analyses were performed using R software (Version 4.4.2; R Foundation for Statistical Computing).

### Ethical aspects

Institutional review board approval (IRB 2025‐0624) was obtained from the participating institution, and the requirement for informed consent was waived owing to the retrospective nature of the study.

## RESULTS

### Cohort and group distribution

Overall, 263 robotic‐assisted primary TKAs with preoperative flexion contracture were included in the analytic cohort. Based on preoperative flexion contracture measured under anaesthesia at the initial robotic assessment, 160 knees (60.8%) were in the >0°–5° group, 74 (28.1%) were in the >5°–10° group and 29 (11.0%) were in the >10° group. Final intraoperative outcomes were available for 227 knees (139, 63 and 25, respectively). Paired preoperative and 1‐year postoperative KOOS‐JR scores were available for 137 knees (88, 34 and 15, respectively). Implant type differed across groups, with the proportion of PS implants increasing with contracture severity (CR: 91.2%, 82.4% and 65.5%; PS: 8.8%, 17.6% and 34.5% in the >0°–5°, >5°–10° and >10° groups, respectively; *p* = 0.001). In knees with final intraoperative outcome data, alignment‐specific findings are shown in Tables S1–S3.

### Baseline characteristics and pre‐resection intraoperative mechanics

Baseline demographic and radiographic characteristics were generally similar across flexion contracture groups (Table 1). Preoperative mechanical HKA angle did not differ among groups (*p* = 0.854), nor did sex distribution (*p* = 0.224), BMI (*p* = 0.194) or Kellgren–Lawrence grade distribution (*p* = 0.140). Nine knees were graded as Kellgren–Lawrence Grade 1 on standing radiographs; in these cases, the indication for arthroplasty was based on the overall clinical and imaging assessment rather than Kellgren–Lawrence grade alone. Implant type differed across groups (*p* = 0.001), with PS implants used more frequently in the >10° group.

**Table 1:** Demographics, radiographic alignment, range of motion and pre‐resection balance metrics stratified by flexion contracture group.

Variable	>0°–5°	>5°–10°	>10°	*p* value
	*n* = 160	*n* = 74	*n* = 29	
Age, years	69.1 ± 8.3	70.7 ± 8.4	69.4 ± 7.5	0.428
	Range, 49–86	Range, 43–89	Range, 56–82	
Sex, female, *n* (%)	93, 58.1%	47, 63.5%	13, 44.8%	0.224
BMI, kg/m^2^	31.9 ± 6.0	33.2 ± 7.0	33.6 ± 5.2	0.194
	Range, 18.9–49.4	Range, 20.8–53.4	Range, 26.8–48.9	
Laterality, right, *n* (%)	76, 47.5%	46, 62.2%	14, 48.3%	0.105
Kellgren–Lawrence grade, *n* (%)				0.140
Grade 1	9, 5.6%	0, 0.0%	0, 0.0%	
Grade 2	58, 36.3%	22, 29.7%	9, 31.0%	
Grade 3	77, 48.1%	47, 63.5%	16, 55.2%	
Grade 4	16, 10.0%	5, 6.8%	4, 13.8%	
Preoperative HKA angle degree	4.6 ± 9.1	3.9 ± 9.2	4.6 ± 9.3	0.854
	Range, −21.2 to 21.0	Range, −15.4 to 20.8	Range, −15.8 to 23.8	
Pre‐resection maximum extension angle degree	2.5 ± 1.5	7.1 ± 1.4	12.9 ± 2.0	<0.001
	Range, 0.1–5.0	Range, 5.1–10.0	Range, 10.4–17.3	
Pre‐resection maximum flexion angle degree	112.2 ± 11.5	111.3 ± 10.2	109.5 ± 10.5	0.419
	Range, 90.8–147.5	Range, 90.8–141.3	Range, 86.7–130.4	
Final maximum extension angle degree	−1.2 ± 4.0	1.1± 4.2	0.9 ± 4.9	<0.001
	Range, −14.2 to 11.1	Range, −8.6 to 11.9	Range, −6.1 to 11.5	
Final maximum flexion angle degree	114.1 ± 14.1	112.9 ± 12.8	110.4 ± 15.2	0.448
	Range, 90.1–177.1	Range, 91.3–138.1	Range, 64.6–136.9	
Preoperative extension angle degree	1.0 ± 2.8	1.7 ± 2.9	4.7 ± 4.5	<0.001
	Range, −5 to 20	Range, −5 to 10	Range, 0–15	
Preoperative flexion angle degree	109.5 ± 10.7	107.0 ± 10.1	101.9 ± 7.8	<0.001
	Range, 90–140	Range, 85–130	Range, 90–120	
Postoperative 6‐week extension angle degree	1.2 ± 2.1	1.0 ± 1.6	2.5 ± 2.8	0.030
	Range, −5 to 10	Range, −2 to 5	Range, 0–10	
Postoperative 6‐week flexion angle degree	109.5 ± 10.3	106.0 ± 13.2	107.9 ± 9.8	0.138
	Range, 80–135	Range, 30–130	Range, 80–120	
Residual flexion contracture >5° at 6 weeks, *n* (%)	4, 2.5%	0, 0.0%	3, 10.3%	0.024
Pre‐resection extension gap, medial, mm	3.7 ± 2.0	3.0 ± 1.9	2.4 ± 1.7	0.001
	Range, 0.0–10.1	Range, 0.0–8.8	Range, 0.0–5.2	
Pre‐resection extension gap, lateral, mm	5.3 ± 2.1	5.5 ± 1.9	6.1 ± 2.4	0.226
	Range, 0.3–11.9	Range, 1.1–10.2	Range, 1.8–11.7	
Pre‐resection extension gap imbalance (lateral−medial), mm	1.6 ± 3.2	2.5 ± 2.9	3.7 ± 3.4	0.005
	Range, −5.6 to 11.9	Range, −5.1 to 8.7	Range, −1.8 to 11.7	
Pre‐resection coronal laxity range under stress in extension degree	10.6 ± 2.3	9.5 ± 1.8	8.0 ± 1.8	<0.001
	Range, 5.3–19.2	Range, 5.5–14.8	Range, 5.2–11.2	
Implant type, *n* (%)				0.001
CR	146, 91.2%	61, 82.4%	19, 65.5%	
PS	14, 8.8%	13, 17.6%	10, 34.5%	
Manipulation under anaesthesia within 1 year, *n* (%)	14, 4.9%	2, 2.7%	3, 10.3%	0.244

*Note*: Varus HKA is positive and valgus is negative. Coronal laxity range under stress was defined as varus minus valgus excursion. Gap imbalance was defined as lateral minus medial (positive values indicate a relatively looser lateral compartment).

Abbreviations: BMI, body mass index; CR, cruciate‐retaining; HKA, hip–knee–ankle; PS, posterior‐stabilized.

Before bone resection, increasing flexion contracture severity was associated with a progressively tighter medial extension gap (3.7 ± 2.0 vs. 3.0 ± 1.9 vs. 2.4 ± 1.7 mm; *p* = 0.001), whereas the lateral extension gap did not differ (*p* = 0.226). Extension gap imbalance increased with contracture severity (1.6 ± 3.2, 2.5 ± 2.9 and 3.7 ± 3.4 mm; *p* = 0.005), while coronal laxity range under manual stress in extension decreased (10.6 ± 2.3°, 9.5 ± 1.8° and 8.0 ± 1.8°; *p* < 0.001).

After adjustment, these findings remained materially unchanged. Compared with the >0°–5° group, the >10° group had a smaller pre‐resection medial extension gap (*β* = −1.29 mm, *p* = 0.001) and a smaller pre‐resection coronal laxity range in extension (*β* = −2.57°, *p* < 0.001).

### Clinical range of motion

Preoperative extension and flexion angles differed across groups (both *p* < 0.001). At 6 weeks postoperatively, extension differed across groups (1.2 ± 2.1°, 1.0 ± 1.6° and 2.5 ± 2.8°, *p* = 0.030), whereas flexion did not (109.5 ± 10.3°, 106.0 ± 13.2° and 107.9 ± 9.8°, *p* = 0.138). Residual flexion contracture >5° at 6 weeks was observed in 4 knees (2.5%), 0 knees (0.0%) and 3 knees (10.3%), respectively (*p* = 0.024). Manipulation under anaesthesia (MUA) within 1 year occurred in 14 knees (4.9%), 2 knees (2.7%) and 3 knees (10.3%), respectively (*p* = 0.244).

In adjusted analyses, the between‐group difference in 6‐week extension remained significant for the >10° group (*β* = 1.33°, *p* = 0.002), whereas 6‐week flexion remained similar across groups.

### Resection strategy, coronal resection angles, tibial slope and tibial‐side offset

**Figure 3:**
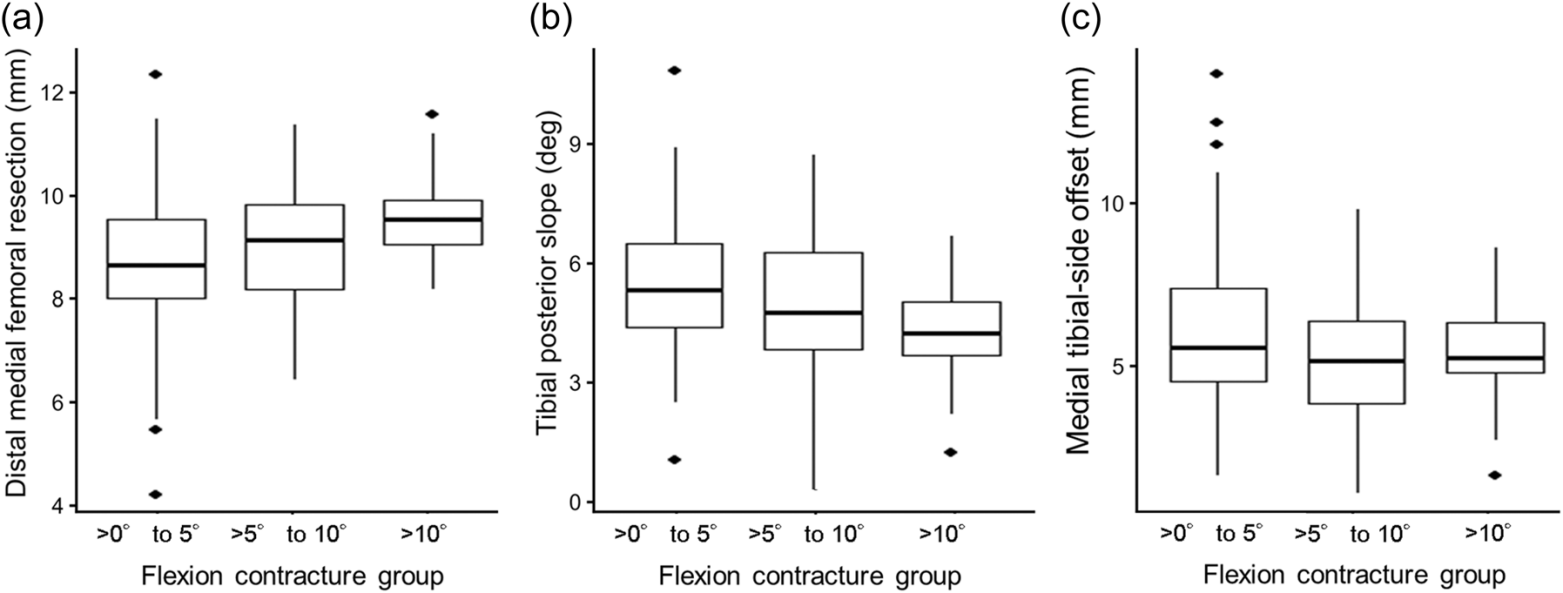
Box‐and‐whisker plots comparing (a) distal medial femoral resection depth (mm), (b) tibial posterior slope (degrees) and (c) medial tibial‐side offset (mm) across flexion contracture groups (>0°–5°, >5°–10° and >10°). Medial tibial‐side offset was defined as the tibial construct thickness minus the validated medial proximal tibial resection depth; higher values indicate a relatively more proximal tibial joint line.

In the 227 knees with complete final intraoperative measurements, distal medial femoral resection increased with flexion contracture severity (9.0 ± 1.3 vs. 9.3 ± 1.2 vs. 9.8 ± 0.8 mm; *p *< 0.001) (Table 2). Distal lateral femoral resection did not differ significantly across groups (*p* = 0.359). Medial proximal tibial resection showed a marginal difference across groups (*p* = 0.050), whereas lateral proximal tibial resection and posterior femoral resections did not differ significantly. Tibial construct thickness remained similar across groups (*p* = 0.908), and femoral and tibial coronal resection angles were also comparable. Tibial posterior slope decreased slightly with increasing contracture severity (5.6 ± 1.6°, 5.1 ± 1.7° and 4.6 ± 1.3°; *p *= 0.002). Medial and lateral tibial‐side measures did not differ significantly across groups (Figure 3).

**Table 2:** Intraoperative resections and final gap/coronal laxity outcomes by flexion contracture group.

Variable	>0°–5°	>5°–10°	>10°	*p* value
	*n* = 139	*n* = 63	*n* = 25	
Distal femoral resection, medial, mm	9.0 ± 1.3	9.3 ± 1.2	9.8 ± 0.8	<0.001
	Range, 4.3–12.7	Range, 6.6–11.7	Range, 8.4–11.9	
Distal femoral resection, lateral, mm	7.6 ± 1.9	7.6 ± 2.0	8.3 ± 2.4	0.359
	Range, 2.9–11.7	Range, 2.0–11.3	Range, 4.5–13.2	
Posterior femoral resection, medial, mm	9.6 ± 1.6	9.4 ± 1.3	10.2 ± 1.5	0.098
	Range, 3.9–12.6	Range, 5.8–12.9	Range, 7.1–13.1	
Posterior femoral resection, lateral, mm	7.3 ± 2.0	7.6 ± 2.1	8.1 ± 2.0	0.182
	Range, 0.1–11.5	Range, 3.1–12.3	Range, 4.2–12.1	
Proximal tibial resection, medial, mm	4.7 ± 1.8	5.3 ± 1.7	5.2 ± 1.4	0.050
	Range, −3.0 to 8.4	Range, 0.8–8.8	Range, 1.9–8.3	
Proximal tibial resection, lateral, mm	6.3 ± 2.1	6.3 ± 2.1	5.4 ± 2.2	0.140
	Range, −1.9 to 11.4	Range, 0.4–10.1	Range, −0.3 to 9.5	
Distal femoral coronal resection angle degree	−0.3 ± 0.9	−0.3 ± 1.1	−0.4 ± 1.0	0.908
	Range, −2.4 to 2.0	Range, −2.9 to 2.3	Range, −2.1 to 1.7	
Tibial coronal resection angle degree	1.2 ± 1.3	0.8 ± 1.1	1.3 ± 1.8	0.129
	Range, −1.9 to 4.0	Range, −2.2 to 3.4	Range, −2.8 to 4.9	
Tibial posterior slope degree	5.6 ± 1.6	5.1 ± 1.7	4.6 ± 1.3	0.002
	Range, 1.1–11.2	Range, 0.3–9.0	Range, 1.3–6.9	
Tibial construct thickness (polyethylene plus baseplate), mm	10.3 ± 0.6	10.3 ± 0.7	10.4 ± 0.8	0.908
	Range, 10.0–12.0	Range, 10.0–13.0	Range, 10.0–13.0	
Medial tibial‐side offset, mm (tibial construct thickness—medial tibial resection depth)	5.6 ± 1.9	5.1 ± 1.7	5.1 ± 1.4	0.083
	Range, 1.7–13.0	Range, 1.2–9.2	Range, 1.7–8.1	
Lateral tibial‐side offset, mm (tibial construct thickness—lateral tibial resection depth)	4.0 ± 2.3	4.0 ± 2.0	4.9 ± 2.7	0.231
	Range, −1.4 to 11.9	Range, −0.1 to 9.6	Range, 0.5–13.3	
Final extension gap, medial, mm	2.6 ± 1.2	2.7 ± 1.3	2.7 ± 1.0	0.977
	Range, 0.8–9.0	Range, 0.9–7.6	Range, 1.4–5.8	
Final extension gap, lateral, mm	3.2 ± 1.4	3.2 ± 1.8	2.8 ± 0.9	0.235
	Range, 1.0–10.1	Range, 1.1–11.7	Range, 1.6–4.7	
Final extension gap imbalance (lateral − medial), mm	0.5 ± 1.2	0.5 ± 1.3	0.1 ± 1.0	0.266
	Range, −3.6 to 5.8	Range, −3.3 to 4.1	Range, −1.7 to 1.9	
Final flexion gap (90°), medial, mm	2.6 ± 2.0	2.5 ± 1.8	1.9 ± 1.4	0.125
	Range, 0.3–17.7	Range, 0.5–9.7	Range, 0.0–5.1	
Final flexion gap (90°), lateral, mm	3.4 ± 2.5	3.2 ± 1.9	2.8 ± 2.1	0.346
	Range, 0.4–20.9	Range, 0.7–8.8	Range, 0.0–6.6	
Final flexion gap imbalance (lateral−medial), mm	0.9 ± 1.8	0.7 ± 1.4	0.9 ± 1.6	0.734
	Range, −3.9 to 6.3	Range, −4.0 to 4.3	Range, −1.6 to 5.6	
Final coronal laxity range under stress in extension degree	4.4 ± 1.4	4.3 ± 1.5	4.1 ± 0.9	0.512
	Range, 1.4–10.4	Range, 1.3–8.1	Range, 2.5–5.8	
Final coronal laxity range under stress in flexion degree	5.3 ± 3.8	4.7 ± 3.9	4.2 ± 3.8	0.315
	Range, 0.2–14.6	Range, 0.2–13.4	Range, 0.0–11.5	
Final maximum extension angle degree	−0.5 ± 3.8	1.2± 4.2	0.9 ± 4.9	0.030
	Range, −14.2 to 11.1	Range, −8.6 to 11.9	Range, −6.1 to 11.5	
Final maximum flexion angle degree	113.1 ± 15.1	112.9 ± 12.8	110.4 ± 15.2	0.687
	Range, 90.1–177.1	Range, 91.3–138.1	Range, 64.6‐136.9	

*Note*: All resections and gap measurements are reported in millimetres, and all angular measurements are reported in degrees. The tibial‐side offset was calculated as the tibial construct thickness (polyethylene plus baseplate) minus validated proximal tibial resection depth for each compartment. Gap imbalance was defined as lateral minus medial values, with positive values indicating a relatively looser lateral compartment. Coronal laxity range under stress was defined as varus excursion minus valgus excursion.

After adjustment, distal medial femoral resection remained greater in the >10° group (*β* = 0.83 mm, *p* = 0.002), whereas tibial posterior slope remained lower in the >10° group (*β* = −0.65°, *p* = 0.049). Tibial construct thickness remained similar across groups after adjustment.

### Final intraoperative gaps, coronal laxity range and range of motion

Final medial and lateral gaps in extension and at 90° flexion were similar across flexion contracture groups (Table 2). Final extension and flexion gap imbalances, as well as coronal laxity range under stress in extension and flexion, also did not differ across groups. Final maximum extension angle differed statistically across groups (−0.5 ± 3.8°, 1.1 ± 4.2° and 0.9 ± 4.9°; *p* = 0.030) but remained near neutral on average, whereas final maximum flexion angle did not differ (*p* = 0.687). In exploratory analyses stratified by preoperative alignment, distal medial femoral resection increased with flexion contracture severity in varus knees (*p* < 0.001), but not in neutral (*p* = 0.663) or valgus knees (*p* = 0.567) (Tables S1–S3).

Adjusted analyses yielded similar results, with no significant between‐group differences in final extension gaps, final flexion gaps or final coronal laxity ranges.

### KOOS‐JR outcomes

In the subset with paired preoperative and 1‐year postoperative KOOS‐JR scores (*n* = 137), 88 knees were in the >0°–5° group, 34 in the >5°–10° group and 15 in the >10° group. Preoperative, postoperative and change in KOOS‐JR scores did not differ across groups (*p* = 0.336, *p* = 0.696 and *p *= 0.231, respectively). These findings were unchanged in adjusted analyses, with no significant between‐group differences in preoperative KOOS‐JR, postoperative KOOS‐JR or KOOS‐JR change scores (Table 3).

**Table 3:** Preoperative and postoperative KOOS‐JR and change scores stratified by flexion contracture group.

Variable	>0°–5°	>5°–10°	>10°	*p* value
	*n* = 88	*n* = 34	*n* = 15	
KOOS‐JR (preoperative)	52.6 ± 16.4	49.6 ± 10.9	48.2 ± 11.2	0.336
	Range, 0.0–84.6	Range, 31.3–70.7	Range, 20.9–61.6	
KOOS‐JR (postoperative)	80.1 ± 17.5	82.6 ± 14.0	79.9 ± 12.9	0.696
	Range, 0.0–100.0	Range, 42.3–100.0	Range, 50.0–100.0	
KOOS‐JR change (post−pre)	27.5 ± 20.3	32.9 ± 13.6	28.5 ± 11.4	0.231
	Range, −36.9 to 92.0	Range, 0.0–53.3	Range, −11.6 to 47.7	

Abbreviation: KOOS‐JR, Knee injury and Osteoarthritis Outcome Score for Joint Replacement.

### Continuous association between flexion contracture severity and distal medial femoral resection

**Figure 4:**
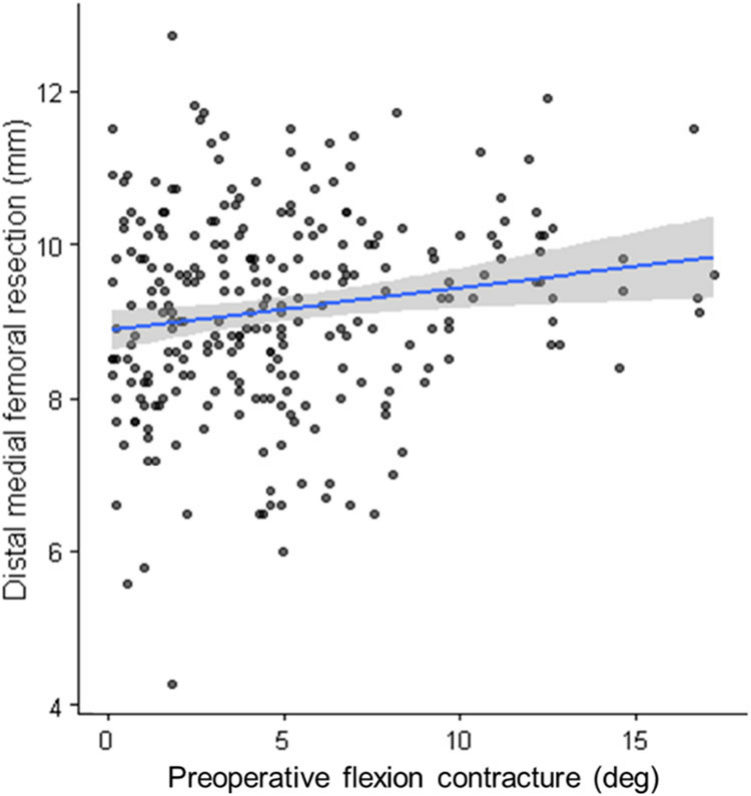
Scatter plot showing the association between preoperative flexion contracture severity and distal medial femoral resection in knees with preoperative flexion contracture. The solid line represents the fitted linear regression line, and the shaded area indicates the 95% confidence interval for the mean predicted resection depth. Each point represents a single knee.

When flexion contracture severity was analysed as a continuous variable among knees with preoperative flexion contracture, each additional degree of flexion contracture was associated with greater distal medial femoral resection (*β* = 0.06 mm per degree, *p *= 0.009) (Figure 4).

## DISCUSSION

The most important finding of this study was that increasing preoperative flexion contracture was associated with only minor differences in intraoperative resection strategy, particularly distal medial femoral resection, whereas final intraoperative balance and early outcomes were broadly similar across groups. Tibial‐side adjustments were limited, and tibial construct thickness remained essentially unchanged across flexion contracture groups. Despite substantial differences in pre‐resection stiffness and extension imbalance, final intraoperative gaps and coronal laxity range were comparable across groups. Early clinical outcomes, including 6‐week range of motion and 1‐year KOOS‐JR scores, also did not differ meaningfully between groups.

Additional distal femoral resection has traditionally been used to increase the extension gap when correcting flexion contracture, often described as a 2–4 mm recut [7, 9, 21, 23]. However, this pattern was not observed in the present cohort. Distal medial femoral resection increased with contracture severity, but the absolute difference was small, at approximately 0.9 mm between the >0°–5° and >10° groups. In contrast, distal lateral femoral resection did not differ significantly between groups. Accordingly, these findings should not be interpreted as indicating a uniform increase in distal femoral resection with increasing flexion contracture. Rather, the observed asymmetry may reflect workflow‐specific balancing adjustments within this surgical technique. Consistent with this interpretation, when flexion contracture severity was analysed as a continuous variable among knees with preoperative flexion contracture, each additional degree was associated with only a 0.06‐mm increase in distal medial femoral resection. The small effect size is consistent with prior studies, suggesting that the gain in terminal extension attributable to distal femoral resection is limited and supporting the view that correction of flexion contracture is not achieved through distal resection alone [9]. Similarly, the observed difference in tibial posterior slope was also small in absolute magnitude. Importantly, both the femoral and tibial differences should be interpreted cautiously within the context of this workflow. Instead, correction is likely multifactorial, reflecting the combined effects of osteophyte removal, posterior capsular release and selective soft‐tissue balancing [13, 21]. Within a robotic workflow, these findings suggest that flexion contracture can be managed without reliance on large distal recuts or substantial tibial modification, which may otherwise raise concern for joint‐line elevation and mid‐flexion laxity [5, 7].

A notable finding of this study was that tibial construct thickness (polyethylene plus baseplate) remained stable across flexion contracture severities [4, 12]. The consistent use of tibial constructs, predominantly between 10 and 12 mm, reflects the reproducibility of this balancing approach in the present cohort. Additional tibial‐side modifications were limited, consisting primarily of a small increase in medial tibial resection thickness and a slight reduction in posterior tibial slope (absolute difference <1° between groups). As patients with flexion contractures often present with normal flexion, a gap imbalance typically exists, characterized by a tight extension gap and normal flexion gap. One way to address this gap imbalance is to enlarge the extension gap with minimal additional distal femoral resection (0.06 mm per degree), increase medial tibial resection and reduce tibial slope to decrease the flexion gap. This concept, known as gap convergence (enlarging the extension gap while reducing the flexion gap), has been previously described [19]. In addition, flexion contracture is a sagittal plane deformity that may occur across a range of coronal plane alignments. Accordingly, tibial and distal femoral coronal resection angles did not differ by contracture severity and remained close to neutral, suggesting that correction was not achieved by systematically cutting either the tibia or distal femur into greater varus in stiffer knees.

Medial tibial‐side offset (construct thickness minus validated tibial resection) decreased slightly with increasing contracture, whereas lateral offset did not differ. This finding suggests the absence of systematic tibial joint‐line elevation; this indirect metric reflects the balance between tibial resection and insert thickness captured by the robotic platform [2, 10].

The most clinically relevant finding was that final intraoperative balance metrics were consistent across flexion contracture severities. Knees with greater preoperative flexion contracture, at initial intraoperative assessment, exhibited a tighter medial extension gap, greater extension imbalance and reduced coronal laxity, reflecting a stiffer pre‐release extension gap compared with less contracted knees. Final maximum extension differed significantly across groups but remained near neutral on average. The influence of flexion contracture severity on these final intraoperative outcomes was not significant in multivariable linear regression models, and results were consistent when severity was analysed as a continuous variable among knees with preoperative flexion contracture. Collectively, these findings suggest that robotic‐assisted workflows can help normalize intraoperative balance targets across a wide spectrum of preoperative stiffness profiles [11, 24].

In the subset of knees with paired patient‐reported outcome measures (PROMs), 1‐year KOOS‐JR scores and improvements were similar across flexion contracture groups [8, 14]. This analysis was limited by PROM availability and sample size, particularly in the most severe contracture group; however, the distribution of contracture severity in the KOOS‐JR subset was similar to that of the overall cohort. These findings align with the intraoperative results, indicating that comparable balance metrics were achieved without detectable differences in early patient‐reported function. KOOS‐JR scores improved at 1 year across all groups.

This study has some limitations. First, the retrospective, single‐surgeon design may limit generalizability. Second, analyses of final intraoperative outcomes were restricted to knees with complete final intraoperative measurements, as these variables were unavailable in the exported robotic record for some cases. This approach may have introduced selection bias if knees with missing data differed systematically from those with complete data. Third, patient‐reported outcomes were unavailable for a subset of knees, introducing the possibility of attrition and selection bias in the KOOS‐JR analyses. Fourth, although preoperative and 6‐week postoperative range‐of‐motion data, including residual flexion contracture >5° at 6 weeks, were available and analysed, 1‐year postoperative range‐of‐motion data were not consistently recorded. Fifth, implant design was selected intraoperatively based on ligamentous stability and balancing requirements, which may have introduced selection bias. Sixth, surgeon‐applied stress testing may have varied in force and positioning, and flexion contracture measured under anaesthesia may not fully reflect awake clinical extension. Finally, this study was conducted using a single robotic platform and workflow, which may limit generalizability to other systems or techniques.

## CONCLUSIONS

Preoperative flexion contracture was associated with minor differences in intraoperative resection strategy, particularly distal medial femoral resection. Final intraoperative balance and early outcomes were broadly similar across flexion contracture groups.

## AUTHOR CONTRIBUTIONS

**Yugo Morita**: Conceptualization; data curation; formal analysis; methodology; writing—original draft. **Jonggu Shin**: Writing—review and editing. **Mohammed El‐Hassan**: Data curation; writing—review and editing. **Ittai Shichman**: Writing—review and editing. **William J. Long**: Writing—review and editing. **Peter K. Sculco**: Conceptualization; project administration; resources; supervision; writing—review and editing.

## FUNDING INFORMATION

The authors have no funding to report.

## CONFLICT OF INTEREST STATEMENT

William J. Long reports royalties from Zimmer Biomet and Elsevier; speakers bureau and paid presentations for Zimmer Biomet, Sylke and Convatec; stock or stock options in Sylke; service on the editorial or governing board of the *Journal of Arthroplasty*; and board or committee appointments with the Hip Society, the Knee Society and the American Joint Registry Steering Committee. Peter K. Sculco reports royalties from Enovis; speakers bureau/paid presentations for Zimmer; paid consultancy for Enovis and Zimmer; stock or stock options in Intellijoint Surgical and Parvizi Surgical Innovation; research support from Intellijoint Surgical as a principal investigator and a board or committee appointment with *HSS Journal*.

## ETHICS STATEMENT

Institutional review board approval (IRB 2025‐0624) was obtained from the participating institution. Informed consent was waived owing to the retrospective nature of the study.

## Supporting information


**Supplementary Table 1.** Intraoperative Resections and Final Gap/Coronal Laxity Outcomes by Flexion Contracture Group in Varus Knees;
**Supplementary Table 2.** Intraoperative Resections and Final Gap/Coronal Laxity Outcomes by Flexion Contracture Group in Neutral Knees;
**Supplementary Table 3.** Intraoperative Resections and Final Gap/Coronal Laxity Outcomes by Flexion Contracture Group in Valgus Knees.

## Data Availability

The data that support the findings of this study are available on request from the corresponding author. The data are not publicly available due to privacy or ethical restrictions.
