# Supplementary material for: Intraoperative management of pre‐operative flexion contractures using robotic‐assisted total knee arthroplasty
Source: J Exp Orthop. 2026 Jul 16;13(3):e70834. doi: 10.1002/jeo2.70834 (PMC13373939; doi:10.1002/jeo2.70834)
Supplement: Supplementary file 1 — Supplementary Table 1. Intraoperative Resections and Final Gap/Coronal Laxity Outcomes by Flexion Contracture Group in Varus Knees; Supplementary Table 2. Intraoperative Resections and Final Gap/Coronal Laxity Outcomes by Flexion Contracture Group in Neutral Knees; Supplementary Table 3. Intraoperative Resections and Final Gap/Coronal Laxity Outcomes by Flexion Contracture Group in Valgus Knees. [file JEO2-13-e70834-s001.docx]

**Supplementary Table 1.** Intraoperative Resections and Final Gap/Coronal Laxity Outcomes by Flexion Contracture Group in Varus Knees

| Variable | Minimal  ≤5°  n = 156 | Moderate  >5° to 10°  n = 38 | Severe  >10°  n = 16 | *p*-value |
| --- | --- | --- | --- | --- |
| Distal femoral resection, medial, mm | 8.6 ± 1.2  Range, 4.3–11.3 | 9.0 ± 1.4  Range, 6.6–11.7 | 9.7 ± 0.9  Range, 8.4–11.9 | <.001 |
| Distal femoral resection, lateral, mm | 8.0 ± 1.6  Range, 2.9–11.7 | 8.4 ± 1.5  Range, 5.4–11.0 | 9.1 ± 2.0  Range, 6.0–13.2 | .059 |
| Posterior femoral resection, medial, mm | 9.7 ± 1.4  Range, 5.8–12.4 | 9.6 ± 1.2  Range, 7.3–12.9 | 10.4 ± 1.5  Range, 7.1–13.1 | .192 |
| Posterior femoral resection, lateral, mm | 7.4 ± 1.9  Range, 2.7–11.5 | 7.5 ± 2.1  Range, 3.9–12.3 | 8.3 ± 2.1  Range, 4.2–12.1 | .240 |
| Proximal tibial resection, medial, mm | 3.9 ± 2.0  Range, -3.0–8.3 | 4.6 ± 1.5  Range, 1.9–8.4 | 5.0 ± 1.7  Range, 1.9–8.3 | .014 |
| Proximal tibial resection, lateral, mm | 7.0 ± 1.5  Range, 2.9–11.4 | 7.1 ± 1.4  Range, 4.5–10.1 | 6.2 ± 1.7  Range, 2.4–9.5 | .164 |
| Distal femoral coronal resection angle, degrees | 0.2 ± 0.9  Range, -1.7–2.6 | 0.0 ± 1.1  Range, -2.9–2.3 | 0.1 ± 0.9  Range, -1.1–1.7 | .662 |
| Tibial coronal resection angle, degrees | 1.5 ± 1.1  Range, -1.7–4.8 | 1.2 ± 1.2  Range, -2.2–3.4 | 1.9 ± 1.8  Range, -2.8–4.9 | .161 |
| Tibial posterior slope, degrees | 5.6 ± 1.6  Range, 2.3–11.2 | 5.2 ± 1.7  Range, 0.3–8.3 | 4.5 ± 1.5  Range, 1.3–6.9 | .025 |
| Tibial construct thickness (polyethylene plus baseplate), mm | 10.4 ± 0.7  Range, 10.0–13.0 | 10.4 ± 0.8  Range, 10.0–13.0 | 10.1 ± 0.3  Range, 10.0–11.0 | .065 |
| Medial tibial-side offset, mm (tibial construct thickness − medial tibial resection depth) | 6.4 ± 2.2  Range, 2.7–14.5 | 5.7 ± 1.5  Range, 2.9–8.2 | 5.1 ± 1.6  Range, 1.7–8.1 | .006 |
| Lateral tibial-side offset, mm (tibial construct thickness − lateral tibial resection depth) | 3.4 ± 1.7  Range, -1.4–9.3 | 3.2 ± 1.4  Range, -0.1–5.5 | 3.9 ± 1.7  Range, 0.5–7.6 | .364 |
| Final extension gap, medial, mm | 2.3 ± 1.1  Range, 0.8–9.0 | 2.7 ± 1.5  Range, 0.9–7.6 | 2.4 ± 0.7  Range, 1.4–4.0 | .424 |
| Final extension gap, lateral, mm | 3.1 ± 1.3  Range, 0.9–10.1 | 3.6 ± 2.1  Range, 1.1–11.7 | 2.6 ± 0.8  Range, 1.6–4.4 | .044 |
| Final extension gap imbalance (lateral−medial), mm | 0.8 ± 1.1  Range, -3.6–4.9 | 0.9 ± 1.2  Range, -1.1–4.1 | 0.2 ± 1.1  Range, -1.5–1.9 | .113 |
| Final flexion gap (90°), medial, mm | 2.2 ± 1.4  Range, 0.3–6.5 | 2.5 ± 1.8  Range, 0.5–9.7 | 1.5 ± 1.0  Range, 0.0–3.3 | .022 |
| Final flexion gap (90°), lateral, mm | 3.0 ± 1.9  Range, 0.4–8.0 | 3.5 ± 2.0  Range, 0.7–8.8 | 2.3 ± 1.9  Range, 0.0–6.6 | .104 |
| Final flexion gap imbalance (lateral−medial), mm | 0.8 ± 1.6  Range, -3.1–6.3 | 1.0 ± 1.4  Range, -2.0–4.3 | 0.8 ± 1.5  Range, -0.4–5.6 | .66 |
| Final coronal laxity range under stress in extension, degrees | 4.1 ± 1.4  Range, 0.7–7.8 | 4.4 ± 1.7  Range, 1.3–8.1 | 3.8 ± 0.8  Range, 2.5–5.3 | .229 |
| Final coronal laxity range under stress in flexion, degrees | 4.6 ± 3.6  Range, 0.2–12.7 | 4.8 ± 4.2  Range, 0.2–13.4 | 3.3 ± 3.2  Range, 0.0–8.6 | .28 |
| Final maximum extension angle, degrees | -1.1 ± 4.2  Range, -14.2–11.1 | 2.3 ± 4.2  Range, -5.5–11.9 | 0.9 ± 5.0  Range, -6.1–9.6 | <.001 |
| Final maximum flexion angle, degrees | 115.1 ± 14.4  Range, 91.5–177.1 | 111.3 ± 13.3  Range, 91.3–137.7 | 110.9 ± 16.7  Range, 64.6–136.9 | .251 |

All resections and gaps are reported in millimeters, and angles are reported in degrees. The tibial joint-line index was calculated as polyethylene insert thickness minus validated proximal tibial resection depth for each compartment. Gap imbalance was defined as lateral minus medial (positive values indicate a relatively looser lateral compartment). Coronal laxity range under stress was defined as varus excursion minus valgus excursion.

**Supplementary Table 2.** Intraoperative Resections and Final Gap/Coronal Laxity Outcomes by Flexion Contracture Group in Neutral Knees

| Variable | Minimal  ≤5°  n = 34 | Moderate  >5° to 10°  n = 5 | Severe  >10°  n = 3 | *p*-value |
| --- | --- | --- | --- | --- |
| Distal femoral resection, medial, mm | 9.5 ± 1.3  Range, 6.1–12.7 | 9.9 ± 0.6  Range, 8.8–10.4 | 10.6 ± 1.2  Range, 9.3–11.5 | .330 |
| Distal femoral resection, lateral, mm | 7.0 ± 1.8  Range, 1.6–10.5 | 7.0 ± 1.3  Range, 4.8–8.2 | 8.8 ± 3.7  Range, 4.6–11.7 | .761 |
| Posterior femoral resection, medial, mm | 9.3 ± 1.7  Range, 5.2–12.2 | 10.0 ± 1.5  Range, 8.2–12.3 | 9.7 ± 0.4  Range, 9.3–10.1 | .555 |
| Posterior femoral resection, lateral, mm | 7.3 ± 1.9  Range, 3.6–10.3 | 9.1 ± 1.9  Range, 6.4–10.7 | 9.0 ± 0.3  Range, 8.8–9.3 | .003 |
| Proximal tibial resection, medial, mm | 5.1 ± 1.5  Range, 1.8–8.9 | 5.1 ± 2.7  Range, 0.8–7.7 | 5.0 ± 0.2  Range, 4.8–5.1 | .859 |
| Proximal tibial resection, lateral, mm | 6.2 ± 2.0  Range, 1.7–10.0 | 4.8 ± 3.7  Range, 0.4–10.0 | 3.3 ± 0.5  Range, 2.8–3.8 | <.001 |
| Distal femoral coronal resection angle, degrees | -0.5 ± 1.0  Range, -2.1–2.0 | 0.2 ± 1.4  Range, -1.8–1.7 | -1.0 ± 0.5  Range, -1.5–-0.6 | .187 |
| Tibial coronal resection angle, degrees | 0.6 ± 1.3  Range, -2.3–3.7 | 0.9 ± 0.8  Range, -0.3–1.8 | 1.1 ± 0.7  Range, 0.4–1.7 | .607 |
| Tibial posterior slope, degrees | 5.1 ± 1.4  Range, 1.1–8.6 | 4.7 ± 1.5  Range, 3.4–7.3 | 4.8 ± 0.4  Range, 4.4–5.2 | .681 |
| Tibial construct thickness (polyethylene plus baseplate), mm | 10.2 ± 0.7  Range, 10.0–14.0 | 10.0 ± 0.0  Range, 10.0–10.0 | 10.7 ± 1.2  Range, 10.0–12.0 | - |
| Medial tibial-side offset, mm (tibial construct thickness − medial tibial resection depth) | 5.1 ± 1.4  Range, 1.5–8.2 | 4.9 ± 2.7  Range, 2.3–9.2 | 5.7 ± 1.0  Range, 5.0–6.9 | .685 |
| Lateral tibial-side offset, mm (tibial construct thickness − lateral tibial resection depth) | 4.0 ± 2.0  Range, 0.0–9.3 | 5.2 ± 3.7  Range, 0.0–9.6 | 7.4 ± 0.8  Range, 6.7–8.2 | .003 |
| Final extension gap, medial, mm | 2.6 ± 1.0  Range, 1.3–6.8 | 2.8 ± 1.0  Range, 1.1–3.7 | 2.4 ± 1.0  Range, 1.6–3.5 | .879 |
| Final extension gap, lateral, mm | 2.8 ± 1.1  Range, 1.0–4.6 | 3.4 ± 1.4  Range, 1.7–4.9 | 2.4 ± 0.6  Range, 1.8–2.9 | .463 |
| Final extension gap imbalance (lateral−medial), mm | 0.2 ± 1.2  Range, -2.2–2.0 | 0.6 ± 0.8  Range, -0.7–1.4 | 0.0 ± 0.9  Range, -0.9–0.8 | .678 |
| Final flexion gap (90°), medial, mm | 2.2 ± 1.6  Range, 0.4–5.8 | 2.1 ± 1.0  Range, 0.7–3.0 | 2.0 ± 1.3  Range, 0.8–3.3 | .968 |
| Final flexion gap (90°), lateral, mm | 3.1 ± 2.0  Range, 0.6–7.2 | 2.2 ± 1.3  Range, 0.9–4.0 | 3.6 ± 2.5  Range, 1.1–6.1 | .489 |
| Final flexion gap imbalance (lateral−medial), mm | 0.9 ± 1.7  Range, -3.2–5.9 | 0.1 ± 1.2  Range, -1.7–1.6 | 1.6 ± 2.2  Range, 0.3–4.1 | .471 |
| Final coronal laxity range under stress in extension, degrees | 4.0 ± 1.1  Range, 1.5–5.6 | 3.5 ± 1.0  Range, 2.4–4.7 | 4.1 ± 0.9  Range, 3.4–5.1 | .645 |
| Final coronal laxity range under stress in flexion, degrees | 4.9 ± 4.1  Range, 0.3–13.7 | 2.5 ± 2.2  Range, 0.3–5.2 | 5.1 ± 4.0  Range, 0.8–8.6 | .258 |
| Final maximum extension angle, degrees | -1.1 ± 3.6  Range, -8.8–4.5 | -1.9 ± 1.6  Range, -4.1– -0.2 | 0.8 ± 5.7  Range, -5.7–4.8 | .620 |
| Final maximum flexion angle, degrees | 114.0 ± 15.9  Range, 90.1–161.3 | 110.2 ± 8.2  Range, 102.0–122.8 | 113.5 ± 9.5  Range, 106.8–124.4 | .747 |

All resections and gaps are reported in millimeters, and angles are reported in degrees. The tibial joint-line index was calculated as polyethylene insert thickness minus validated proximal tibial resection depth for each compartment. Gap imbalance was defined as lateral minus medial (positive values indicate a relatively looser lateral compartment). Coronal laxity range under stress was defined as varus excursion minus valgus excursion.

**Supplementary Table 3.** Intraoperative Resections and Final Gap/Coronal Laxity Outcomes by Flexion Contracture Group in Valgus Knees

| Variable | Minimal  ≤5°  n = 58 | Moderate  >5° to 10°  n = 20 | Severe  >10°  n = 6 | *p*-value |
| --- | --- | --- | --- | --- |
| Distal femoral resection, medial, mm | 9.4 ± 1.1  Range, 7.3–11.8 | 9.6 ± 0.9  Range, 7.9–11.5 | 9.8 ± 0.4  Range, 9.3–10.4 | .192 |
| Distal femoral resection, lateral, mm | 5.0 ± 2.2  Range, -0.7–9.2 | 6.1 ± 2.2  Range, 2.0–11.3 | 5.9 ± 1.1  Range, 4.5–7.4 | .103 |
| Posterior femoral resection, medial, mm | 9.2 ± 1.7  Range, 3.9–12.6 | 8.9 ± 1.4  Range, 5.8–11.4 | 9.8 ± 1.6  Range, 7.4–11.7 | .547 |
| Posterior femoral resection, lateral, mm | 7.2 ± 2.1  Range, 0.1–11.1 | 7.3 ± 2.0  Range, 3.1–10.3 | 6.8 ± 1.7  Range, 4.6–8.5 | .802 |
| Proximal tibial resection, medial, mm | 5.6 ± 1.2  Range, 2.8–8.6 | 6.4 ± 1.2  Range, 4.2–8.8 | 6.0 ± 0.7  Range, 5.4–7.0 | .048 |
| Proximal tibial resection, lateral, mm | 3.9 ± 2.3  Range, -1.9–9.4 | 5.2 ± 2.0  Range, 1.7–9.3 | 4.4 ± 2.8  Range, -0.3–8.4 | .116 |
| Distal femoral coronal resection angle, degrees | -0.9 ± 0.7  Range, -2.4–0.5 | -0.9 ± 0.9  Range, -2.4–0.6 | -1.2 ± 1.0  Range, -2.1–-0.5 | .813 |
| Tibial coronal resection angle, degrees | -0.2 ± 0.8  Range, -1.9–1.8 | 0.1 ± 0.8  Range, -1.3–1.4 | -0.2 ± 1.0  Range, -1.4–1.6 | .343 |
| Tibial posterior slope, degrees | 5.4 ± 1.4  Range, 2.6–9.2 | 5.0 ± 1.8  Range, 2.4–9.0 | 4.7 ± 1.0  Range, 3.5–6.3 | .313 |
| Tibial construct thickness (polyethylene plus baseplate), mm | 10.4 ± 0.8  Range, 10.0–14.0 | 10.4 ± 0.8  Range, 10.0–13.0 | 10.8 ± 1.3  Range, 10.0–13.0 | .758 |
| Medial tibial-side offset, mm (tibial construct thickness − medial tibial resection depth) | 4.8 ± 1.3  Range, 1.7–7.5 | 4.0 ± 1.3  Range, 1.2–6.8 | 4.8 ± 1.0  Range, 3.3–6.0 | .081 |
| Lateral tibial-side offset, mm (tibial construct thickness − lateral tibial resection depth) | 6.5 ± 2.6  Range, 0.6–14.6 | 5.2 ± 1.9  Range, 2.3–9.3 | 6.4 ± 4.0  Range, 1.6–13.3 | .116 |
| Final extension gap, medial, mm | 3.3 ± 1.0  Range, 1.3–6.4 | 2.7 ± 1.2  Range, 1.3–6.0 | 3.5 ± 1.5  Range, 1.4–5.8 | .212 |
| Final extension gap, lateral, mm | 3.1 ± 1.3  Range, 1.0–7.6 | 2.5 ± 1.0  Range, 1.3–5.0 | 3.5 ± 1.1  Range, 1.7–4.7 | .062 |
| Final extension gap imbalance (lateral−medial), mm | -0.2 ± 1.4  Range, -2.2–5.8 | -0.2 ± 1.1  Range, -3.3–1.5 | -0.0 ± 1.0  Range, -1.7–1.3 | .878 |
| Final flexion gap (90°), medial, mm | 3.0 ± 3.2  Range, 0.0–18.5 | 2.6 ± 1.8  Range, 0.7–6.8 | 3.0 ± 1.8  Range, 0.9–5.1 | .795 |
| Final flexion gap (90°), lateral, mm | 4.3 ± 3.8  Range, 0.0–21.9 | 3.0 ± 1.6  Range, 0.8–6.0 | 3.6 ± 2.4  Range, 0.9–6.6 | .128 |
| Final flexion gap imbalance (lateral−medial), mm | 1.3 ± 1.8  Range, -3.9–6.4 | 0.3 ± 1.5  Range, -4.0–2.4 | 0.7 ± 1.8  Range, -1.6–3.8 | .101 |
| Final coronal laxity range under stress in extension, degrees | 4.9 ± 1.5  Range, 0.7–10.4 | 4.4 ± 1.0  Range, 2.1–5.8 | 5.0 ± 0.8  Range, 3.7–5.8 | .223 |
| Final coronal laxity range under stress in flexion, degrees | 5.6 ± 3.8  Range, 0.0–14.6 | 5.0 ± 3.5  Range, 0.3–9.6 | 6.3 ± 4.8  Range, 0.4–11.5 | .733 |
| Final maximum extension angle, degrees | -1.4 ± 3.7  Range, -10.2–8.4 | -0.3 ± 3.9  Range, -8.6–5.9 | 0.9 ± 5.5  Range, -4.9–11.5 | .407 |
| Final maximum flexion angle, degrees | 111.7 ± 12.1  Range, 90.7–134.9 | 116.6 ± 12.3  Range, 100.0–138.1 | 107.5 ± 14.7 Range, 92.4–126.6 | .258 |

All resections and gaps are reported in millimeters, and angles are reported in degrees. The tibial joint-line index was calculated as polyethylene insert thickness minus validated proximal tibial resection depth for each compartment. Gap imbalance was defined as lateral minus medial (positive values indicate a relatively looser lateral compartment). Coronal laxity range under stress was defined as varus excursion minus valgus excursion.
